# Cardiovascular effects of *Ekebergia capensis* Sparrm (Meliaceae) ethanolic leaf extract in experimental animal paradigms

**Published:** 2009-05

**Authors:** David R Kamadyaapa, Mavuto M Gondwe, Kogi Moodley, Cephas T Musabayane, John AO Ojewole

**Affiliations:** Department of Human Physiology, School of Medical Sciences, University of KwaZulu-Natal, Durban, South Africa; Department of Human Physiology, School of Medical Sciences, University of KwaZulu-Natal, Durban, South Africa; Department of Human Physiology, School of Medical Sciences, University of KwaZulu-Natal, Durban, South Africa; Department of Human Physiology, School of Medical Sciences, University of KwaZulu-Natal, Durban, South Africa; Department of Pharmacology, School of Pharmacy and Pharmacology, University of KwaZulu-Natal, Durban, South Africa

## Abstract

**Summary:**

The purpose of this study was to examine the *in vivo* effects of *Ekebergia capensis* leaf ethanolic extract (EKE) on the blood pressure of anaesthetised normotensive male Wistar rats and conscious weanling Dahl salt-sensitive (DSS) rats, which develop hypertension as they age. To investigate possible mechanism(s) of the extract’s hypotensive effects, the contractile or relaxant responses to EKE in the absence or presence of reference drugs were evaluated in Wistar rat isolated aortic rings precontracted with methoxamine hydrochloride (ME, 10 μM).

Acute intravenous administration of EKE elicited hypotensive responses in anaesthetised animals, while sub-chronic treatment with the extract averted the development of high blood pressure in weanling DSS rats. Isometric recordings of methoxamine hydrochloride (ME) pre-contracted, isolated, endothelium-intact and -denuded aortic rings revealed concentration-dependent relaxation responses to EKE (1–160 mg/ml). The potency was significantly less in the endothelium-denuded rings. Inhibitors of endothelium-derived relaxing factor (EDRF), L-NAME, methylene blue and indomethacin significantly reduced EKE-evoked vasorelaxations in endothelium-intact aortic rings.

These results indicate that the vasorelaxant effect of EKE was in part mediated via EDRF-dependent or -independent pathways. These observations suggest that the hypotensive effect of EKE was in part mediated via modulation of total peripheral resistance of the vascular smooth muscles.

## Summary

The World Health Organisation and many developing countries have significant interest in complimentary health systems, perhaps due to the integral part played by ethnomedicinal plants in folkloric healthcare.^1^ *Ekebergia capensis* Sparrm (Meliaceae), a fairly large tree and widespread in southern Africa, plays an important role in various communities.^2^ For instance, *E capensis* extracts are used for heartburn, coughs and respiratory complaints, and decoctions made from the wood of the plant are used by the Zulus in KwaZulu-Natal as oxytocic agents.^3^,^4^

In spite of the widespread use of *E capensis* extracts in folk medicine and the availability of a reasonable number of scientific observations on its medicinal properties, we could not find any report on its effects on the cardiovascular system. *Ekebergia senegalenensis* A Juss which belongs to the Meliaceae family has, however, been reported to contain bioactive chemical compounds such as glycosides, polyphenols, tannins, triterpenes and saponins.^4^,^5^ We were therefore motivated to examine the effects of *E capensis* leaf extract on the blood pressure of rats, based on the knowledge that *Ekebergia* spp extracts contain glycosides, and the fact that cardiac glycosides used therapeutically to increase cardiac contractility are of plant origin.^6^

The main aim of this study was therefore to assess the effects of *E capensis* leaf extract on blood pressure of normotensive Wistar and weanling genetically hypertensive Dahl salt-sensitive (DSS) rats, which develop hypertension as they age. Since we needed more information about the mechanism(s) of action of the extract, we also evaluated the *in vitro* cardiovascular effects of *E capensis* leaf extract (EKE) on rat isolated atrial muscle strips, and its vasorelaxant effects on isolated thoracic aortic rings and portal veins of normotensive Wistar rats. We envisaged that establishment of the mechanism(s) of its cardiovascular effects would provide scientific evidence for the development of a cheap and accessible source of novel drugs for the treatment of cardiovascular disorders in impoverished, developing populations.

## Materials

The reference drugs used in the present study were: methoxamine hydrochloride (ME), acetylcholine chloride (ACh), indomethacin, N^G^-nitro-L-arginine-methyl-ester (L-NAME), methylene blue, atropine sulphate (ATR), glibenclamide, (±)-propranolol hydrochloride, (-)-noradrenaline hydrochloride (NA), prazosin, reserpine and nifedipine (all from Sigma, St Louis, MO, USA). All chemicals were of the analytical grade and supplied by Merck Chemicals, South Africa.

Indomethacin and glibenclamide were separately dissolved in 0.5% sodium bicarbonate (1 ml) and dimethyl sulphoxide (DMSO, 1 ml), respectively, and deionised water (19 ml) before use. All other drug solutions, including Kreb-Henseleit solution (KHS) were freshly prepared in deionised water daily at the beginning of our experiments.

Leaves of *Ekebergia capensis* Sparrm (Meliaceae), identified by Prof H Baijnath, former chief taxonomist/curator of the Department of Botany, University of KwaZulu-Natal were collected on the Westville Campus of the University between January and June 2005. A voucher specimen of the plant has been deposited in the Botany Department Herbarium.

Normotensive (normal) male Wistar (250–300 g) and weanling Dahl salt-sensitive rats (100–150 g) bred and housed at the Biomedical Research Unit, University of KwaZulu-Natal were used in this study. The rats were maintained on a 12-h light/12-h dark regime, and given both food (Epol diet 4700, Epol, South Africa) and water *ad libitum*. Ethical clearance was obtained for this study from the University Ethics Committee.

## Methods

Ethanolic *E capensis* leaf extracts (1 kg) were prepared as previously described by Musabayane *et al*.^7^ Freeze-drying and solvent elimination under reduced pressure produced 42.85 g of a light brown, powdery leaf extract (EKE), a yield of 4.29%. The crude extract was used without further purification. Aliquot portions of the plant extract residue were weighed and dissolved in deionised water for use on each day of our experiments.

## Whole animal experiments

The acute and chronic effects of EKE on the mean arterial blood pressure (MAP) and heart rate were examined *in vivo* in the Wistar and DSS rats, respectively. The effect of EKE on myocardial contractile performance was evaluated on rat isolated atrial muscle strips, whereas the vasodilatory effects were determined on isolated thoracic aortic rings and portal veins of the Wistar rats. Arterial blood pressure and heart rate were measured in anaesthetised Wistar and conscious DSS rats as previously described by Musabayane *et al*.^7^

**Acute effects of EKE:** Wistar rats anaesthetised by intraperitoneal injection of inactin [(5-ethyl-5-(1’-methylpropyl)-2-thiobarbiturate, 0.11 g/kg body weight (Sigma Aldrich, St Louis, Missouri, USA)] were placed on a thermally controlled heating table (37 ± 1°C). After tracheotomy, a catheter was inserted into the jugular vein for intravenous infusion of 0.077 M NaCl at 9 ml/h (Harvard syringe infusion Pump 22, Harvard Apparatus, Holliston, Massachusetts, USA). An additional heparinised catheter was also inserted into the left carotid artery for blood pressure and heart rate measurement at 30-min intervals via a pressure transducer (Statham MLT 0380, Ad Instruments, Bella Vista NSW, Australia) compatible with PowerLab System ML410/W (Bella Vista NSW, Australia).

Following a 3-h equilibration period, measurements were recorded over the 4-h post-equilibration period of the 1-h control, 1 h 30-min treatment and 1 h 30-min recovery periods. In those animals in which the effects of the extract were studied, EKE was added to the infusate at 360 μg/h for 1.5 h (treatment period), resulting in a total dose of 18 mg/kg (for a 300-g rat), before the animals were returned to infusate alone for the last 1.5 h (recovery period). Depth of anaesthesia was monitored throughout the experiments and additional intravenous bolus doses of inactin (0.05 g/kg body weight) were administered when necessary.

**Chronic effects of EKE:** Mean arterial blood pressure (MAP) and systolic and diastolic pressures were measured every third consecutive day for seven weeks at 09:00, in separate groups of untreated control and EKE-treated (120 mg/kg po) DSS rats (*n* = 8 per group). Control rats were similarly treated with deionised water (3 ml/kg). The cardiovascular effects of EKE were measured by the tail-cuff method with computerised blood pressure monitoring (IITC Model 31 computerised blood pressure monitor, Life Sciences, Woodland Hills, CA). The method was standardised and used routinely in our laboratory as described previously.^7^ Briefly, the rats were subjected to a training programme which involved placing them in restrainers and warm holders, and measuring blood pressure on the tail before the start of the studies.

**Inotropic and chronotropic effects of EKE:** The effects of EKE on myocardial contractile performance were evaluated on rat isolated atrial muscle strips of Wistar rats as previously described.^7^

To evaluate the effects of EKE on the myocardial contractile force (inotropic), electrically driven left atria were impaled on thin platinum wire electrodes and stimulated (5–10 mV) with square wave pulses of 5-ms duration at a frequency of 3 Hz, via an SRI stimulator (Preamplifier, Bioscience, UK). To examine the extract’s effect on atrial pacemaker activity (chronotropic), isolated spontaneously beating right atria of rats were set up under the same experimental conditions. Two isolated electrically driven left atrial muscle strips and two isolated spontaneously beating right atrial muscle preparations were set up each time (one as the test, and the other as the control preparation) to allow for changes in the atrial muscle sensitivity.

Concentration–response curves to EKE (1–40 mg/ml) and/or reference agonist drugs were obtained. Control atrial muscle strips were treated with volumes of deionised water equivalent to the volumes of bath-applied EKE solution (0.1–0.6 ml). The electrically provoked and spontaneous contractions of the atrial muscles, as well as the EKE- and reference agonist drug-induced responses of the muscle preparations were recorded isometrically by means of Ugo Basile force–displacement transducers and pen-writing Gemini recorders (model 7070). The effects of EKE and reference drugs were expressed as percentages of the baseline values (*n* = 8 preparations for each concentration).

## *In vitro* vascular effects of EKE

**Isolated aortic rings:** The effects of EKE on vascular smooth muscles were evaluated using aortic rings isolated from normotensive Wistar rats as described previously.7 Control aortic rings with and without functional endothelium were pre-contracted with a single sub-maximal concentration of methoxamine hydrochloride (ME, 10 μM). Satisfactory removal of the functional endothelium was checked by at least 10% and 70% relaxation, respectively, to 10^-6^ M ACh.

After a sustained, stable, tonic contraction was obtained with ME, concentration-response curves for EKE (1–160 mg/ml) and/or the reference agonist drugs were obtained. The involvement of endothelium-derived relaxing factor in EKE-induced relaxation was examined in intact aortic rings pre-treated with appropriate antagonists [N^G^-nitro-L-arginine methyl ester, L-NAME (100 μM), nitric oxide synthase inhibitor, methylene blue (10 μM), guanylate cyclase inhibitor, and indomethacin (10 μM), a nonselective cyclooxygenase inhibitor].

To assess the role of potassium or calcium in the vasorelaxant effect of the extract, concentration–response curves of EKE were constructed in endothelium-intact aortic rings precontracted with low K^+^ (20 mM) and high K^+^ concentrations (80 mM), respectively, in the presence glibenclamide, as previously described.^8^-^10^ The contractile and/or relaxant effects of all the reference drugs used, as well as EKE-induced relaxations on the aortic ring preparations were recorded isometrically by means of the force–displacement transducers and Gemini recorders.

Isolated portal veins: The Wistar rats were sacrificed and the isolated portal vein preparations were prepared as previously reported,7 and graded concentrations of EKE (2.5, 10 or 40 mg/ml) were added to the bath fluid. To investigate whether the effects of EKE were mediated through modulation of alpha-1 adrenergic receptors or voltage-operated calcium channels, some of the portal vein preparations were pre-treated with either an alpha-1 adrenergic receptor blocker, prazosin (1 μM), or an L-type voltage-operated calcium channel blocker, nifedipine (1 μM), five minutes before re-establishing the cumulative concentration–response curves to EKE.

Control venous muscle strips were treated with deionised water equivalent to the volumes of bath-applied EKE solution. Two isolated venous tissue preparations (one control and the other EKE- or reference drug-treated test) were set up in order to make allowance for changes in the venous tissue sensitivity. The plant extract and/or reference drug-induced responses of the muscle preparations, recorded by means of Gemini recorders, were calculated as percentage of the baseline values (*n* = 8 preparations for each concentration).

## Statistical analysis

Data obtained from test isolated atria, aortic ring strips and portal veins as well as those from control atrial strips and anaesthetised Wistar and conscious DSS rats treated with EKE were pooled and expressed as means ± standard error of means (SEM). Statistical comparison of the differences between treated means (EKE and reference drugs) and control means was performed with GraphPad InStat Software (version 3.00, GraphPad Software, San Diego, California, USA), using one-way analysis of variance (ANOVA; 95% confidence interval), followed by Tukey-Kramer multiple comparison tests. A value of *p* < 0.05 was considered significant.

## Results

## *In vivo* hypotensive effects of EKE

**Fig. 1.**
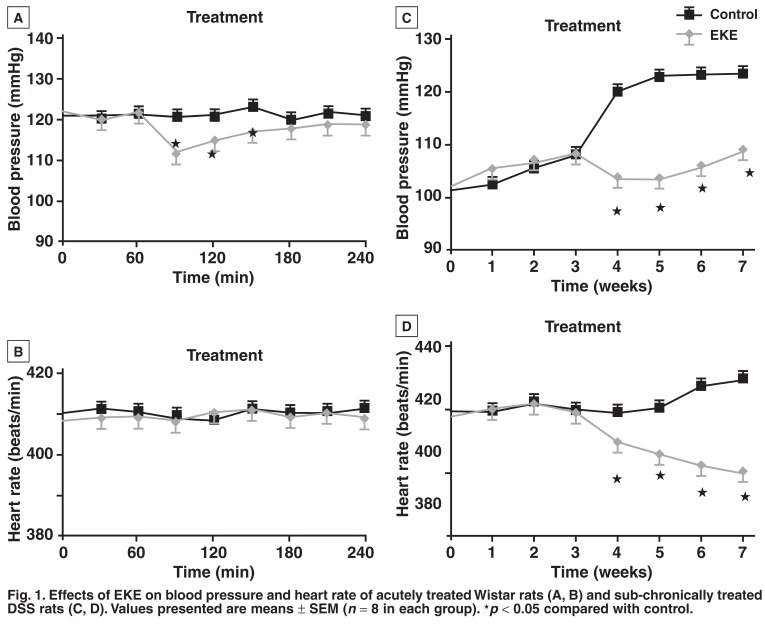
Effects of EKE on blood pressure and heart rate of acutely treated Wistar rats (A, B) and sub-chronically treated DSS rats (C, D). Values presented are means ± SEM (n = 8 in each group). ★*p* < 0.05 compared with control.

Acute intravenous infusion of EKE (360 μg/h) in the normotensive Wistar rats induced a transient fall in MAP within 30 min without a significant effect on the heart rate (Fig. 1A, B). The blood pressure, however, progressively increased and by the end of the treatment period, had reached values comparable with those recorded during the control period in the control animals at the end of the experiment. The results of blood pressure and heart rate monitoring after seven weeks of daily intraperitoneal EKE (80 mg/kg) showed that from the fourth week onwards, EKE prevented the development of hypertension in DSS rats, with no significant effect on the heart rate (Fig. 1C, D).

**Fig. 2.**
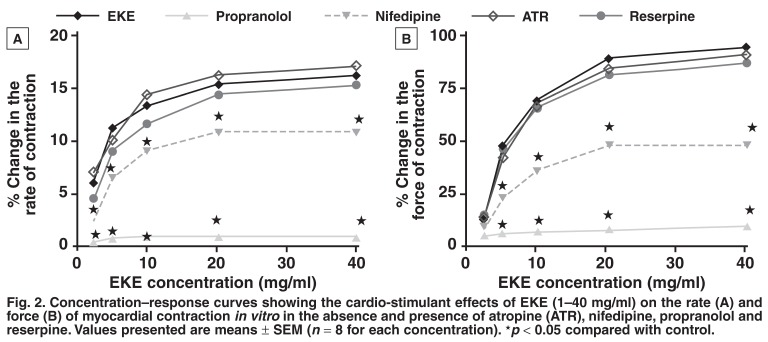
Concentration–response curves showing the cardio-stimulant effects of EKE (1–40 mg/ml) on the rate (A) and force (B) of myocardial contraction in vitro in the absence and presence of atropine (ATR ), nifedipine, propranolol and reserpine. Values presented are means ± SEM (*n* = 8 for each concentration). ★*p* < 0.05 compared with control.

**Isolated atrial muscles:** Sequential administrations to the bath fluid of low to high concentrations of EKE (2.5–40 mg/ml) produced significant (*p* < 0.05), concentration-dependent, positive inotropic and positive chronotropic effects of EKE on isolated electrically driven left and spontaneously beating right atrial muscles taken from the normotensive rats, respectively (Fig. 2A, B). Propranolol (1 μM), a non-selective β-adrenergic receptor antagonist, almost completely inhibited the inotropic and chronotropic effects of EKE (Fig. 2). On the other hand, nifedipine (1 μM), a voltage-operated calcium channel blocker also caused a significant (*p* < 0.05) partial abolition of the positive inotropic and chronotropic effects of EKE (Fig. 2). These effects were, however, not significantly affected by pre-treatment with atropine or reserpine.

**Fig. 3.**
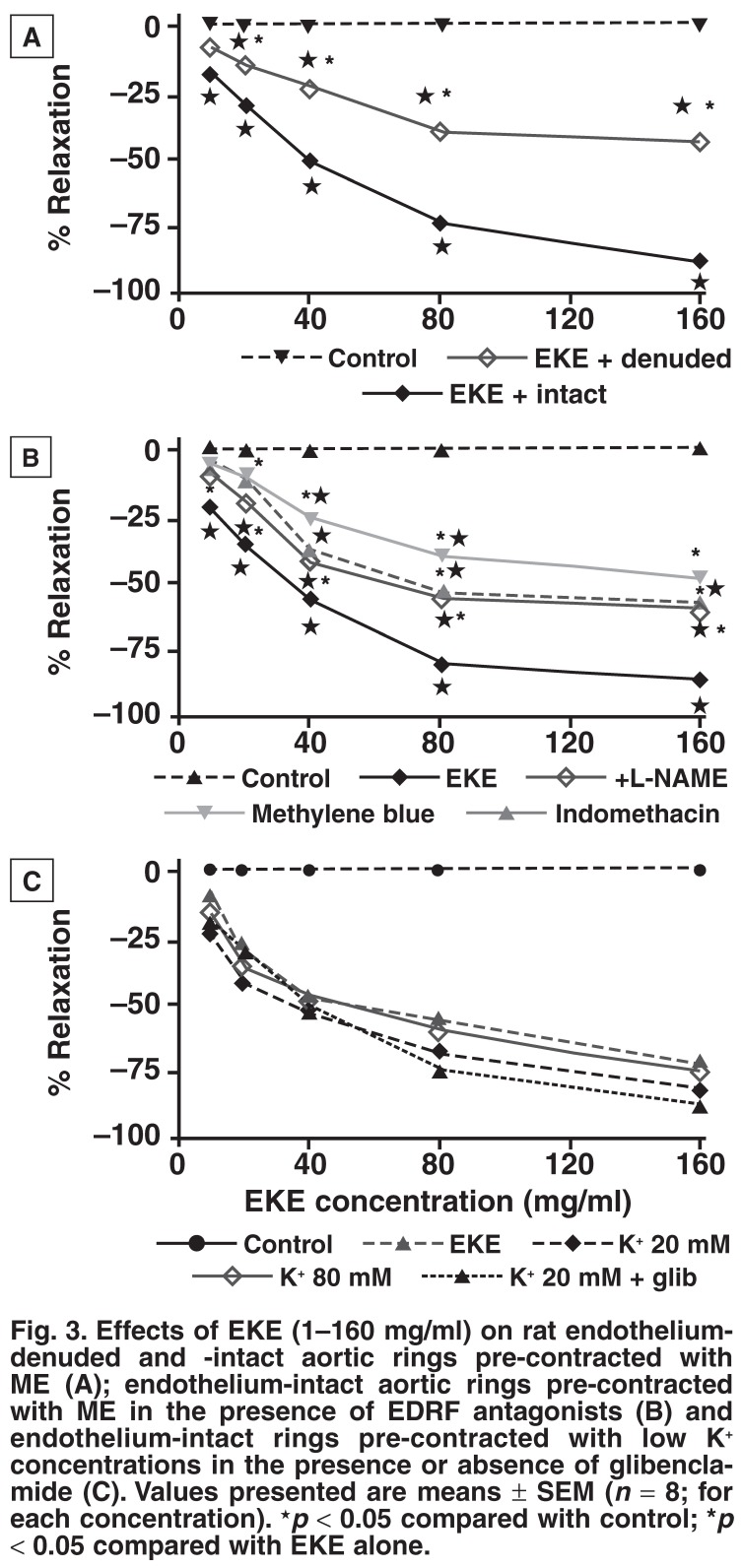
Effects of EKE (1–160 mg/ml) on rat endothelium-denuded and -intact aortic rings pre-contracted with ME (A); endothelium-intact aortic rings pre-contracted with ME in the presence of EDR F antagonists (B) and endothelium-intact rings pre-contracted with low K^+^ concentrations in the presence or absence of glibenclamide (C). Values presented are means ± SEM (*n* = 8; for each concentration). ★*p* < 0.05 compared with control; *p < 0.05 compared with EKE alone.

**Isolated aortic rings:** Exogenous additions of graded concentrations of EKE (1–160 mg/ml) to aortic ring strips pre-contracted with ME, an alpha-1 adrenergic receptor agonist, evoked concentration-dependent relaxation responses of the muscle strips (Fig. 3A). The responses of EKE in endothelium-denuded aortic rings, however, were significantly lower than those with endotheliumintact rings (Fig. 3A). Endothelium-intact preparations were therefore used to study the roles of endothelial-derived relaxing factor, calcium and potassium channels in the vasorelaxant effects of EKE. The vasorelaxant effects of EKE in endotheliumintact aortic rings were significantly reduced by specific inhibitors of endothelium-derived relaxing factor (EDRF) (L-NAME, methylene blue and indomethacin) (Fig. 3B). Overall, these results suggest that the vasorelaxant effect of EKE was in part dependant on EDRF.

To evaluate the role of potassium channels in the vasorelaxant effects of EKE, studies were conducted on endothelium-intact aortic rings pre-contracted with low K+ concentrations (20 mM). EKE induced significant and concentration-dependent vasorelaxations in these aortic rings. Pretreatment with glibenclamide before inducing contraction with low K^+^ did not modify the vasorelaxant effect of EKE (Fig. 3C).

To evaluate the role of calcium channels in the vasorelaxant effects of EKE, experiments were conducted in endotheliumintact aortic rings pre-contracted with high K^+^ concentrations (80 mM). The vasorelaxant effects of graded EKE concentrations were not statistically different in these aortic rings precontracted with low or high K^+^ concentrations (Fig. 3C). Aortic ring preparations contracted with 80 mM K^+^ have been used to study compounds with Ca^2+^ entry-blocking properties.^8^

**Fig. 4.**
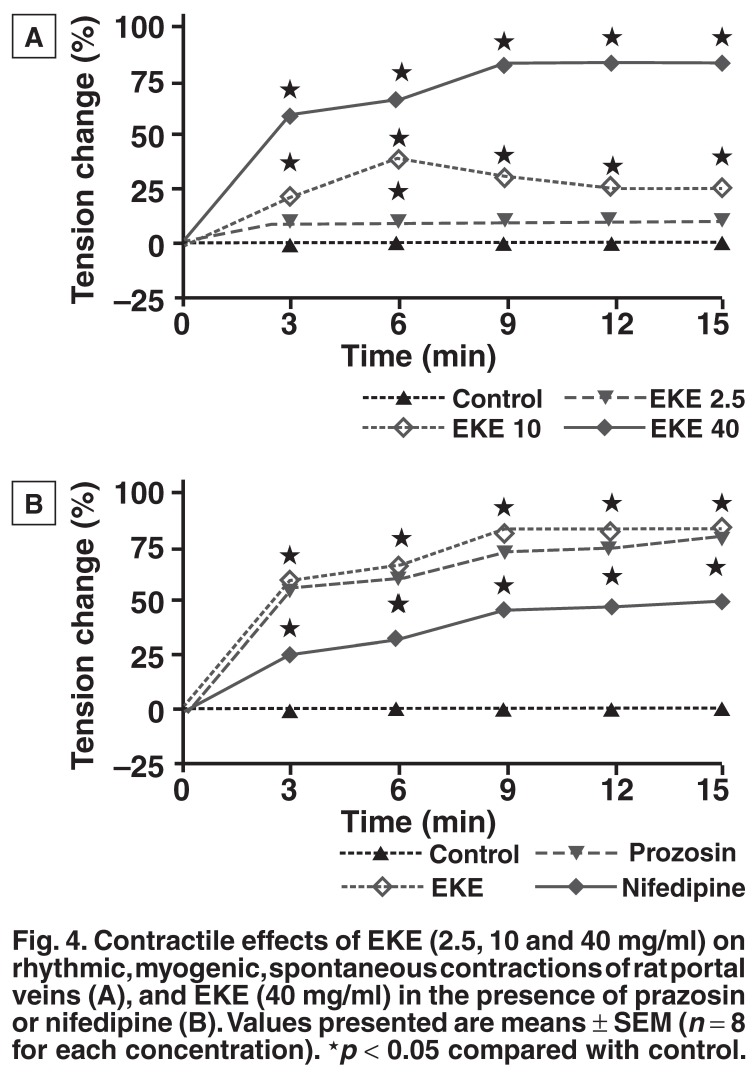
Contractile effects of EKE (2.5, 10 and 40 mg/ml) on rhythmic, myogenic, spontaneous contractions of rat portal veins (A), and EKE (40 mg/ml) in the presence of prazosin or nifedipine (B). Values presented are means ± SEM (*n* = 8 for each concentration). ★*p* < 0.05 compared with control.

**Isolated portal veins:** EKE (2.5, 10 and 40 mg/ml) concentration-dependently increased the amplitude of spontaneous contractions of the rat portal vein preparations isolated from the normotensive rats (Fig. 4A). Nifedipine (1 μM) significantly (*p* < 0.05) reduced the stimulant, contractile effect induced by the high concentration of EKE (40 mg/ml) (Fig. 4B). Pre-treatment of the venous muscle preparations with prazosin (1 μM), however, had no significant effect on EKE-induced stimulant activity (Fig. 4B).

## Discussion

The findings of this study indicate that *E capensis* leaf extract possesses hypotensive properties, since acute and sub-chronic administrations of the extract reduced blood pressure in normotensive Wistar and hypertensive DSS rats, respectively. The DSS rat, which progressively develops hypertension with age, is a genetic rat model of salt-sensitivity hypertension,^11^,^12^ The *in vivo* reduction in blood pressure by the extract occurred without significant alterations in heart rate, possibly suggesting that the *in vitro* cardiovascular effects of EKE significantly contributed to its hypotensive effects. This speculation is corroborated by the fact that the vasorelaxant effects of EKE were demonstrated in isolated vascular smooth muscles (Fig. 3).

The results obtained in this study also suggest that the EKE-evoked vasorelaxations in aortic ring preparations were mediated through both EDRF-dependent and -independent mechanisms. Indeed, graded concentrations of the extract elicited dose-dependent vasorelaxations in endothelium-intact and -denuded aortic ring preparations, although the EKE vasodilatory effect was less in the latter protocol. Furthermore, the vasorelaxations produced by EKE in endothelium-intact aortic rings were pharmacologically modulated by L-NAME, a non-selective nitric oxide synthase inhibitor,^13^ suggesting the involvement of endothelial synthesised nitric oxide (NO).

Endothelial NO synthesis is regulated by a variety of stimuli that trigger release of multiple vasoactive substances, including nitric oxide synthase (NOS).^14^-^17^ NO activates vascular Ca^2+^-activated (Kca) channels directly^18^ and/or through cyclic GMP-dependent mechanisms,^19^ and causes smooth muscle relaxation.^20^-^22^ In the present study, methylene blue, an inhibitor of guanylate cyclase and pharmacological inhibitor of the cyclooxygenase pathway with indomethacin, significantly reduced EKE-elicited vasorelaxations in intact aortic rings. These results suggest that EKE-elicited vasorelaxant effects on the vascular smooth muscles were mediated via NO and/or cGMP and cyclooxygenase pathways. It is now generally accepted that relaxation of vascular smooth muscles involves the lowering of intracellular calcium mediated by cGMP-dependent or -independent pathways.

Removal of the functional aortic endothelium did not completely abolish EKE-evoked vasorelaxations, suggesting the involvement of endothelium-independent relaxing factor. Since EKE concentration dependently evoked vasorelaxations in the endothelium-intact aortic rings pre-contracted with low K^+^ concentrations (20 mM), we speculate that this effect was mediated via endothelium-derived hyperpolarising factor (EDHF), which opens K^+^ voltage-sensitive channels.^20^,^22^ K^+^ depolarises smooth muscle cells to facilitate Ca^2+^ influx, and subsequent vasoconstriction.^20^ Relaxation of smooth muscle contractions induced by low K^+^ concentrations (< 30 mM) are usually mediated via the opening of K_Ca_-activated channels.^23^ Activation of potassium channels, therefore, appears to be another possible mechanism of EKE’s vasorelaxant effects. In blood vessels, several types of Ca^2+^ voltage-sensitive channels participate in the process of excitation–contraction coupling^24^,^25^

EKE produced concentration-dependent vasorelaxation in the endothelium-intact aortic rings pre-contracted with a high K^+^ concentration (80 mM), suggesting the participation of calcium channels in the vasorelaxant effects of the extract. The contractile responses induced by high K^+^ concentrations (80 mM) in K^+^-depolarised muscles are due to the influx of extracellular Ca^2+^ through L-type voltage-sensitive channels (VOCs).^24^ The vasorelaxant effects of EKE against high potassium-induced contractions can therefore be visualised as blockade of Ca^2+^ entry into cells. The vasorelaxant action induced by EKE in K^+^-contracted rings therefore appears to be mediated via inhibition of Ca^2+^ entry, leading to decreased intracellular calcium concentrations. The fact that EKE relaxed pre-contracted endothelium-intact thoracic aortic rings via both endothelium-dependent and -independent mechanisms suggests that the hypotensive action of the extract was elicited, in part at least, by reducing the total peripheral vascular resistance through dilatation of the blood vessels.

The present results suggest that the vasorelaxant effects of EKE in the portal vein preparations were in part mediated via L-type voltage-dependent Ca^2+^ channels, since nifedipine, a Ca^2+^ channel antagonist,^21^,^26^ significantly reduced EKE-elicited increases in the amplitude of the tissue’s spontaneous contractions (Fig. 4A). We excluded the involvement of the α_1_-adrenergic system, since the vasorelaxant effects of EKE were not altered by pre-treatment of the venous muscle preparations with prazosin, an α_1_-adrenergic receptor antagonist.^26^,^27^ Several studies have, however, indicated that contractile activity in portal veins is due to α_1_-adrenergic receptor activation.^26^,^27^

Although the chemical constituents of EKE were not evaluated in this study, compounds reported to be present in the extract include saponins,^28^ alkaloids, flavonoids, tannins and saponosides.^29^ Saponins and polyphenols, including flavonoids have been reported to stimulate nitric oxide release from vascular endothelial cells, and to induce vascular smooth muscle relaxation.^30^-^33^

## Conclusion

This study has provided the mechanistic basis for the use of *E capensis* in the management of hypertension and other cardiovascular disorders. The findings may be helpful in the development of an antihypertensive agent from *E capensis* leaves.
